# Subclinical variability in visual function modulates visual dependence - independent of age

**DOI:** 10.1007/s00221-024-06940-6

**Published:** 2024-10-29

**Authors:** Amir Saman Fathi, David Andrew Green

**Affiliations:** 1 King’s College London, Centre of Human & Applied Physiological Sciences (CHAPS), Room 3.14 Shepherd’s House, Guy’s Campus, London, SE1 1UL UK; 2 Department of Cardiothoracic Surgery, Harefield Hospital, Harefield, UB9 6JH UK; 3 KBR, Wyle Laboratories GmbH, Cologne, Germany; 4 Institute for Risk and Disaster Reduction, University College London, London, WC1E 6BT UK

**Keywords:** Visual dependence, Multisensory integration, Visual function

## Abstract

Paradoxically visual dependence is reported to increase with age, contributing to falls risk, whereas visual function typically declines. This study assesses the relationship between age, objective and subjective measures of visual function and visual dependence, in healthy young and older adults. Forty-four healthy Young (YA; *n* = 32; 18 males, aged 26.2 ± 5.3 yrs.) and Older (OA; *n* = 12; 3 males, aged 62.4 ± 6.7 yrs.) adults were assessed for objective (visual acuity, contrast sensitivity, depth perception, and lower peripheral vision), and subjective visual function (VFQ-25) along with motion sickness susceptibility. Subjective Visual Vertical (SVV) and induced nausea and vection were assessed using the Rod and Disc Test (RDT). Groups were compared using Mann-Whitney U, whilst determinants of SVV variability were evaluated using Multiple regression modelling. Visual acuity (*p* < 0.01) and contrast sensitivity (*p* = 0.04) were lower in OA. Visual dependence (SVV tilt errors) was not associated with ageing (*p* = 0.46). YA experienced greater RDT-induced vection (*p* = 0.03). Visual acuity and contrast sensitivity accounted for modest proportions of variance in SVV tilt errors (VA; R^2^ = 0.14, F(1,42) = 8.00, *p* < 0.01; β = 6.37) and (CS; R^2^ = 0.06, F(1,42) = 3.93, *p* = 0.05; β = −4.97), respectively. Our findings suggest that subclinical differences in visual acuity and contrast sensitivity contribute to SVV tilt error variability, among both healthy young and older adults. Further studies are needed to define the inter-relationship between age-related visual function, non-visual factors (including vestibular and somatosensory fidelity, activity levels, fear of falling and cognitive function) and visual dependence.

## Highlights

• Age alone was not a predictor of visual dependence in healthy individuals.
• Visual acuity and contrast sensitivity were found to be lower in older adults.
• There was no effect of age on vision-related health and quality of life
• Variability in SVV tilt error was associated with sub-clinical differences in visual acuity and contrast sensitivity, rather than age alone.
• There was an indirect effect of ageing on SVV tilt error, mediated by lower visual acuity and contrast sensitivity.

## Introduction

Maintaining spatial orientation and balance (Kaufman et al. 2001) typically require: (i) the integration of vestibular, proprioceptive, and visual signals (Horak 2006), and (ii) a dynamic interplay between compensatory and anticipatory postural adjustments (Santos et al. 2010). In this context, vision holds significant importance, providing dynamic information concerning the body’s position and movement relative to oneself (egocentric framework) (Filimon 2015), as well as contextual information about the surrounding environment, points or frames of reference, and potential sources of instability (allocentric framework) (Wade and Jones 1997).

When visual, vestibular, and/or proprioceptive signals are incongruent, either due to environmental factors and/or sensory processing dysfunction, sensory ‘conflict’ can arise (Keshavarz et al. 2015). Such conflict can result in illusions including the perception of self-motion (i.e., vection)(Berthoz et al. 1975), disorientation, and/or motion sickness (Oman 1990). Typically, the central nervous system attempts to mitigate the impact of sensory conflict through the process of multisensory integration (MSI), with compensatory upregulation of signals from ‘more reliable’ sensory modalities (Peterka 2002; Wallace et al. 2020).

Idiosyncratic upregulation of visual inputs, relative to the other senses, termed visual dependence (VD), (Witkin et al. 1977), is reported to increase with age (Agathos et al. 2015a). The mechanisms underpinning this apparent increase in visual dependence are not well understood but, it is thought to be a sequalae of ageing-related declines in vestibular and/or proprioceptive systems (Osoba et al. 2019a). Inappropriate visual dependence can lead to exaggerated instability in complex visual environments i.e., ‘visual vertigo’ (Cousins et al. 2014), and is associated with an increased risk of falls (Lord and Webster 1990).

Key aspects of visual function namely visual acuity, and contrast sensitivity (Lord and Menz 2000) in addition to peripheral vision (Marigold and Patla 2008), and depth perception (Lord and Dayhew 2001) are reported to decline with age. This decline is significant because, although visual function is crucial for navigation (Turano et al. 2005), poor vision impairs spatial and risk perception, thereby contributing to increased falls risk (Saftari & Kwon, 2018a, b).

The observed increase in visual dependence with age, despite a decline in visual function, represents a paradox. Interestingly, Agathos and colleagues reported an association between age and an increase in visual dependence but did not consider visual function as a confounder (Agathos et al. 2015b). Significant inter-individual variability in visual dependence has been reported in older adults, yet no differences have been observed between visually independent older adults and younger adults (Lee 2017).

More recently, Agathos and Shanidze (2024), reported an association between poor visual function (visual acuity and contrast sensitivity) and increased visual dependence in patients with Central Field Loss (CFL) (Agathos and Shanidze 2024). Notably, the authors posit that CFL does not lead to visual dependence, rather an individual’s degree of visual dependence persists despite loss of visual function – although they tended to have higher visual dependence than a small group of age-matched healthy controls.

Various aspects of objective visual function appear to differentially relate to falls risk (Lord and Webster 1990). Lower subjective vision-related health (e.g., the visual function questionnaire-25; VFQ-25) is associated with reduced quality of life via impact on activities of daily living. However, whether age per se modulates the relationship between objective and/or subjective perception of visual function, and visual dependence (and associated symptoms: vection and nausea) is unknown.

Thus, this study assesses the relationship between age, subjective and objective visual function (visual acuity, contrast sensitivity, depth perception and lower peripheral vision), subjective symptoms and visual dependence in healthy young and older adults.

## Methods

### Participants

Forty-six healthy non-fallers (22 males) were recruited for this study. Participants were recruited from undergraduate and postgraduate university cohorts, as well as among full-time university-educated staff. The study was granted local ethical approval by King’s College London’s ethics committee (BDM/09/10–59) and conformed to the Declaration of Helsinki.

All participants provided written informed consent and completed a comprehensive health screening questionnaire prior to testing. Individuals with major comorbidities or any ophthalmological diseases, apart from corrected myopia or hyperopia (with or without astigmatism), were excluded from the study.

The Young Adult (YA) group (aged 20–40 years) included 32 participants (n = 18 male, 26.2 ± 5.3 yrs.; 173.0 ± 11.0 cm, 66.0 ± 11.8 kg, BMI: 22.0 ± 2.1 kg m^− 2^). The Older Adults (OA) group consisted of twelve participants aged 50–71 years (n = 3 males, 62.4 ± 6.7 yrs., 164.0 ± 7.5 cm, 66.2 ± 10.0 kg, BMI: 24.6 ± 2.9 kg m ^− 2^).

### Experimental design

All participants attended the laboratory on a single occasion for a session lasting 60–90 min. Objective visual function —Visual Acuity (VA) and Contrast Sensitivity (CS)— was assessed monocularly, in a darkened room using participants’ habitual or best corrected vision i.e., glasses/contact lenses. Depth Perception (DP) and Lower Peripheral Vision (LPV) were assessed binocularly in a well-lit room. The levels of Visual Dependence (VD), as indicated by Subjective Visual Vertical (SVV) tilt errors, was conducted inside an environmental chamber that allowed for absolute darkness.

### Subjective assessments

Prior to testing, self-reported visual function status and Quality of Life (QoL) were assessed using the National Eye Institute Visual Function Questionnaire (VFQ-25) (2000 version), consisting of 25 items across 12 domains yielding a composite score (0 to 100). Lower scores are indicative of poor vision-related health and quality of life (Mangione et al. 2001). Situational vertigo (Guerraz et al. 2001) and susceptibility to motion sickness was evaluated via the Motion Sickness Susceptibility Questionnaire (MSSQ) (Golding 1998).

### Objective visual function

Visual Acuity (VA) and Contrast Sensitivity (CS) were assessed using Test Chart 2000 Xpert software (Thomson Software Solutions, UK). VA, measured from six meters, was reported on a Logarithmic scale of Minimum Angle of Resolution (LogMAR; score of ≤ 0.0 corresponds to 6/6 vision or better; a score > 0.3 indicates clinically low VA) (The Royal College of Ophthalmologists 2021). Each letter is assigned a logarithmic value of 0.02, while letter size decreases logarithmically by 0.10 with each row. Testing concluded when all letters on a given row were read incorrectly. VA was reported as LogMAR of the last line where all letters were read correctly, minus any letters identified on subsequent lines. The inability to read any letter from the top line was scored as LogMAR 1.0 (6/60, indicating severe vision loss). The reported VA is an average of the LogMAR scores of the left and right eyes.

Contrast sensitivity was assessed from one meter. Participants were presented with a screen displaying three letters of equal contrast in a single row (a set). Contrast was reduced by a logarithmic (Log_10_) step of 0.15 with each set until participants were no longer able to read any letters. The logarithmic contrast of letters ranged from zero (representing 100% letter contrast) to 2.25 (equivalent to 0.6% letter contrast). Contrast sensitivity was recorded monocularly, as the log contrast of the last line where 2/3 letters were correctly identified, minus the first three letters (100% letter contrast) (Pelli et al. 1988). Here we report the average log contrast of the right and left eyes.

**Fig. 1:**
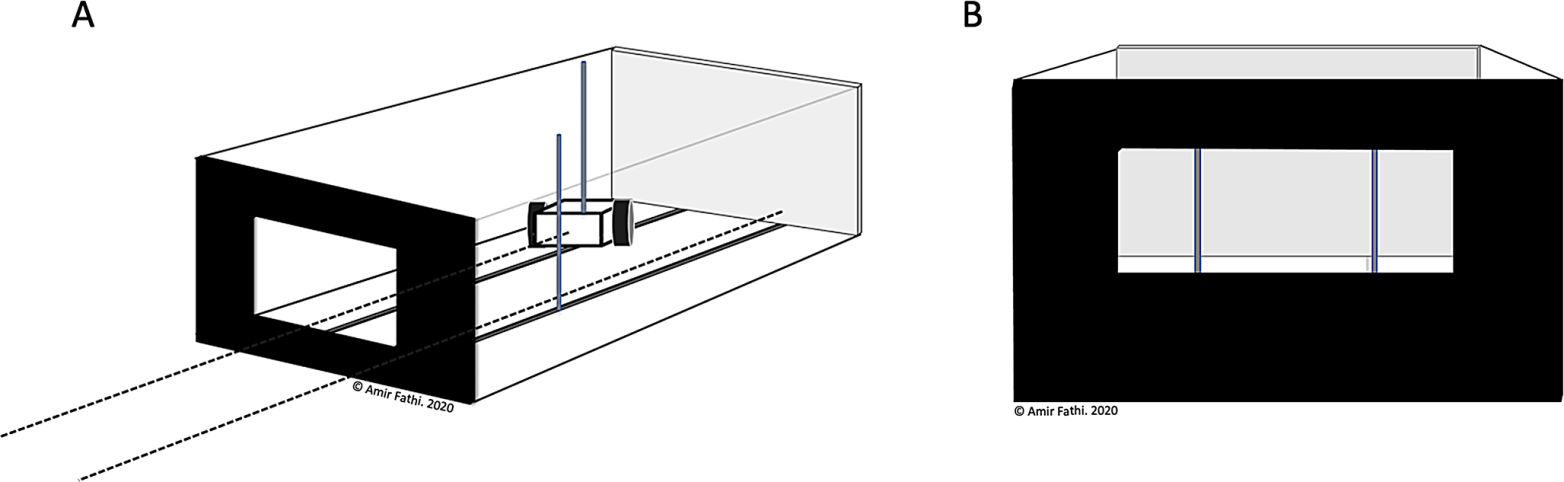
**A**) Schematic of the Howard-Dolman (HD) apparatus used to assess depth perception. Participants used a pulley system (indicated by dashed lines) to adjust a mobile rod (on a carriage) along the Z-axis until it was perceived as aligned with a second stationary rod. Depth perception was reported as the distance (cm) between the two rods (alignment errors). **B**) Anterior view of the HD apparatus window through which participants viewed and aligned the rods

Depth perception (DP) was assessed using the Howard-Dolman apparatus (Howard 1919): a box measuring 63.5 cm length, 29.2 cm width, and 30.5 cm in depth (Fig. 1A), featuring a central window on the anterior surface (Fig. 1B). Two vertical metal rods could be seen from the window: one fixed in the centre, and another mounted onto a mobile carriage, connected via a pulley system. Participants were tasked with re-aligning the two metal rods from six meters until they were perceived as parallel. For each test, the mobile rod was positioned either 15 cm in front, or 15 cm behind the stationary central rod, creating positive and negative errors, respectively.

Binocular depth perception (DP) was recorded as the absolute error (cm), and converted to visual angles (degrees):

$$\:{{\uptheta\:}}_{\text{degrees}}=2\times\:\text{arctan}\left(\frac{d}{2\times\:600}\right)\times\:\left(\frac{180}{{\uppi\:}}\right)$$

where $\:{\theta\:}_{\text{degrees}}\:$ is the visual angle in degrees, *d* represents the error distance (cm), and 600 is the viewing distance in cm. Here, we report the mean visual angle of three trials.

*Lower Peripheral Vision (LPV) testing was performed based on that described by Lord & Menz* (Lord and Menz 2000) *where participants stood upright whilst maintaining a* neutral line of sight with the aid of a height-adjustable, foam padded (to minimise proprioceptive cues) chin guide. Participants, while focusing on a secondary eye-level target, 2.25 m in front of them, were required to confirm sight of a black 4.5 cm radius disc placed on the white floor. *The right*,* left and central visual field boundaries were determined by repositioning the disc horizontally along a straight line*,* starting from the participant’s midline*,* until the disc was no longer visible in the respective visual field.* LPV, recorded in cm, was the furthest distance from the participant’s centre of mass to the last point where the disc was visible. Measurements for the central, right, and left visual fields were averaged with respect to the participant’s eye height from the ground (cm).

### Subjective visual vertical

**Fig. 2:**
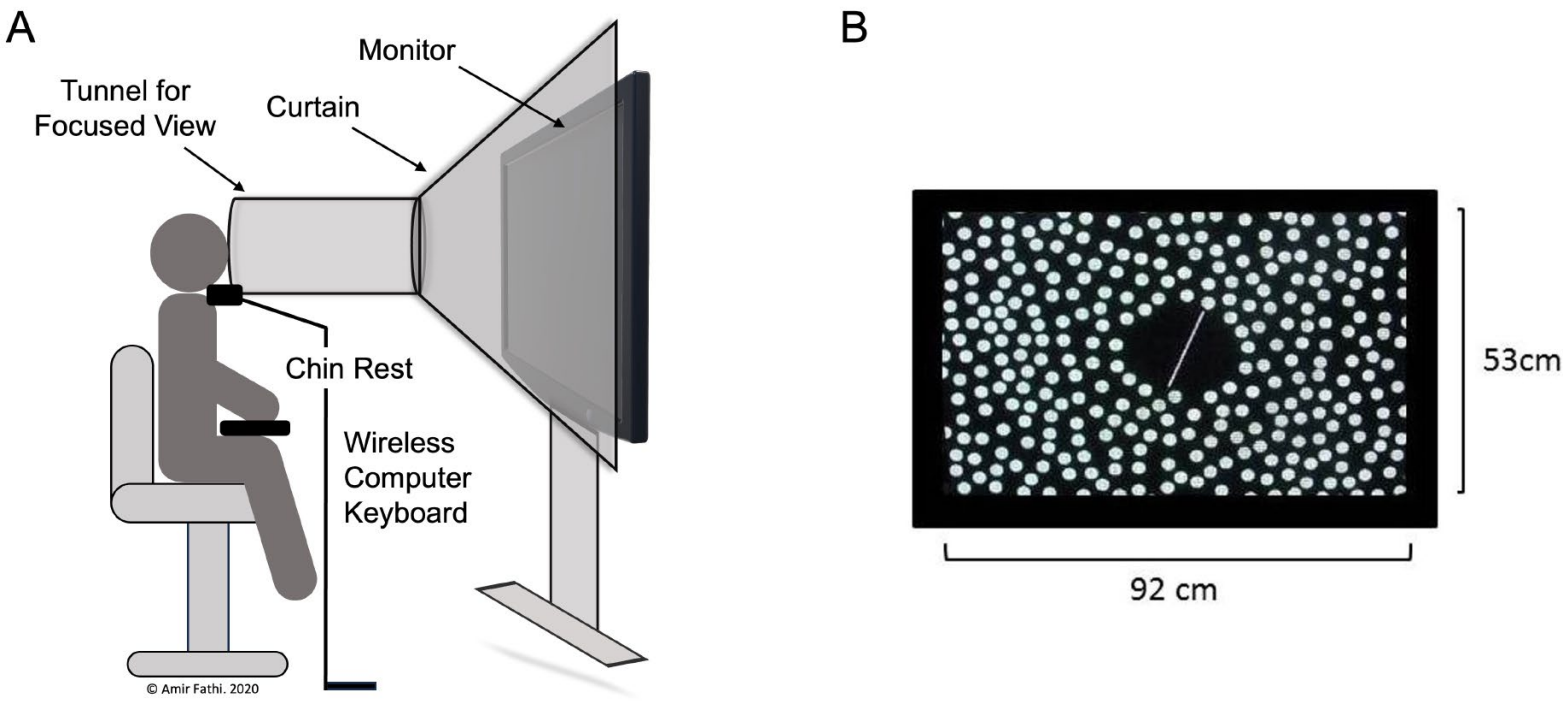
**A**) Setup of the Rod and Disc Test (RDT) used in evaluating Subjective Visual Vertical (SVV) tilt errors, a measure of visual dependence. **B**) Display interface of the RDT, illustrating the observer’s viewpoint during the assessment

Subjective Visual Vertical (SVV) was assessed using the Rod and Disc Test (RDT) (Guerraz et al. 2001) in a darkened environmental chamber, with participants seated 80 cm from a screen (40-inch Panasonic LCD). Participants positioned their head inside a cardboard tunnel, whilst their chin rested on a foam-lined adjustable chin rest, and the feet hanging freely, to minimise proprioceptive cues (Fig. 2A). Vestibular activation was reduced by limiting rotational or tilting head movements.

During the test, participants re-aligned a white, fluorescent rod, superimposed on a black rotating disk (30° s-1), to the true visual vertical, using a keypad (Logitech, UK; Fig. 2B). The fluorescent rod, tilted 20° in a clockwise (CW) or counter-clockwise (CCW) direction, was re-aligned eight time across four trials (totalling 32 re-alignments). Prior to each re-alignment, participants closed their eyes, while the investigator tilted the rod to the right (CW) or left (CCW). Deviations from the true visual vertical (SVV tilt errors) were recorded in degrees and presented as the absolute mean errors. Greater SVV tilt errors correspond to higher visual dependence. *SVV tilt errors typically range from ≈ 2° in healthy individuals* (Guerraz et al. 2001), *with errors of up to 20° indicative of significant vestibular pathology* (Akin and Murnane 2009). *Therefore*,* any apparent SVV tilt errors exceeding 20° were excluded from the analysis.*

Following the test, RDT-associated vection (sensation of self-motion) and nausea were assessed using a numeric scale ranging from 0 (no vection or nausea) to 5 (strong vection inducing the perception of falling or severe nausea).

### Data analysis

Data normality was assessed via Kolmogorov-Smirnov (KS) testing. As some visual function data were not normally distributed, non-parametric statistical testing (Mann-Whitney U test; two-tailed) was performed to compare the age groups. Simple linear regression was used to evaluate the relationship between visual function (subjective and objective), and visual dependence (absolute SVV tilt error). Subsequently, multiple linear regression was applied to evaluate the effect of age as a co-factor between aspects of visual function and visual dependence. Mediation (Average Causal Mediation Effect; ACME) analysis was performed upon visual function indices shown to relate to visual dependence to evaluate whether they modulate the relationship between visual dependence and age. All statistical analysis were performed using the publicly available software RStudio (R version 4.3.2 (2023-10-31); R Core Team, R Foundation for Statistical Computing, Vienna, Austria, 2023)(R Core Team 2023). All data are presented as Mean ± SD. Statistical significance level was set at *p* < 0.05.

## Results

### Visual function

Two participants were excluded from the study, both unable to read the top line of the visual acuity chart: one OA, later diagnosed with right-sided cataract, and one YA with new myopia. Forty-four (21 males) participants successfully completed the study.

**Fig. 3:**
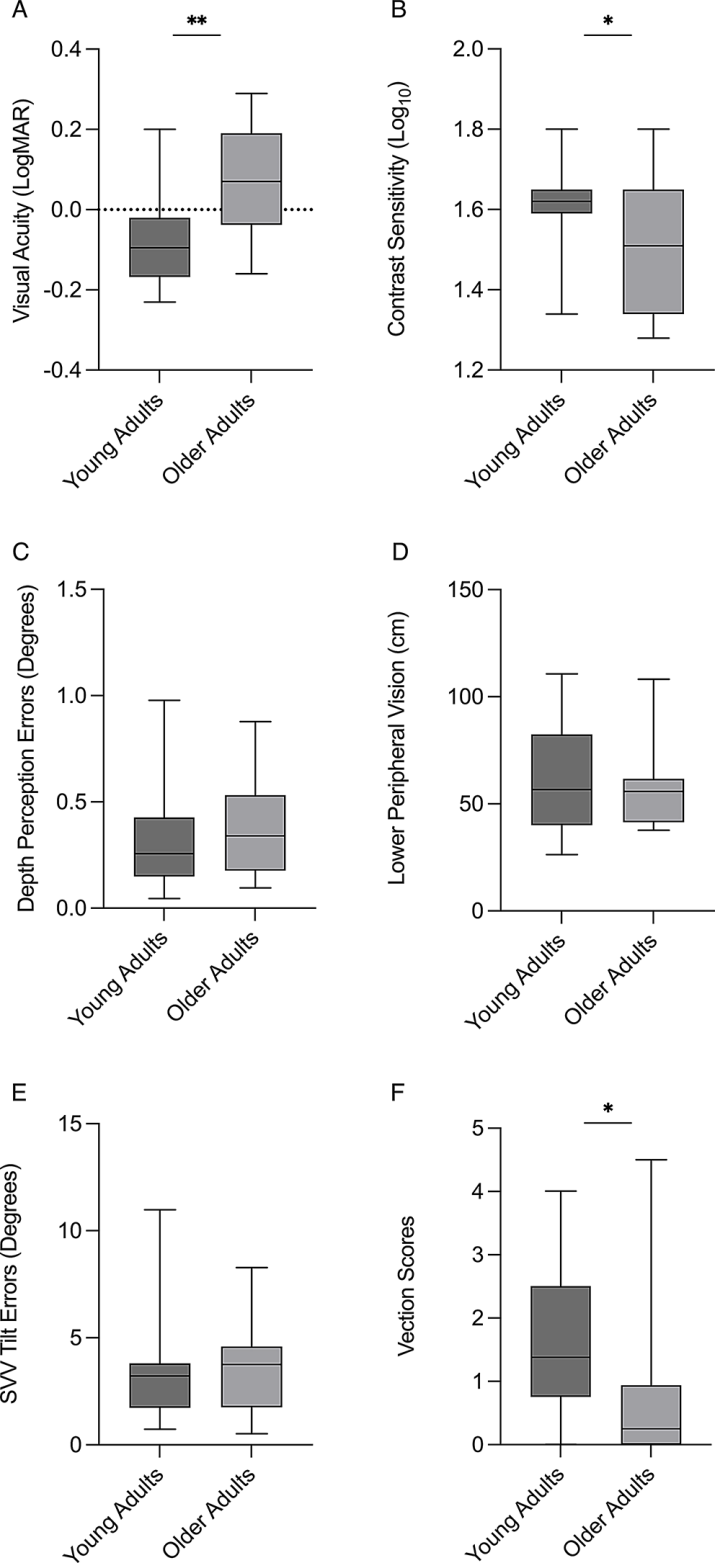
Box and Whisker plots comparing mean scores of young adults (YA) and older adults (OA) for: **A**) Visual Acuity (LogMAR); **B**) Contrast Sensitivity (Log_10_); **C**) Depth Perception alignment errors (Degrees); **D**) Lower Peripheral Visual Field Angle (cm); **E**) Subjective Visual Vertical (SVV) tilt errors (Degrees); and **F**) absolute Vection scores. Asterisks denote level of significance, * = *p* < 0.05; ** = *p* < 0.01

Ten (31%) young adults, and five (42%) older adults required daily refractive visual aids. All participants were found to have clinically good (corrected or habitual) bilateral monocular visual acuity (LogMAR < 0.3). VA (U = 79.0; *p* < 0.01; Fig. 3A) and CS (U = 117.0; *p* = 0.04; Fig. 3B) were lower in the OA group. There were no age-related differences in DP (U = 177.5; *p* = 0.71; Fig. 3C) and LPV (U = 190.5; *p* = 0.98; Fig. 3D).

### Subjective visual vertical (SVV) tilt error

There were no group differences in SVV tilt error (degrees; 3.6 ± 2.2 in OA vs. 3.3 ± 2.1 in YA; U = 163.5; *p* = 0.46; Fig. 3E).

### Subjective assessments

Mean VFQ-25 scores were 91.6 ± 6.3 for OA vs. 94.8 ± 4.5 for YA, showing no significant group differences (U = 130.5; *p* = 0.10). Notably, there was a significant association between VFQ-25 and visual acuity (R^2^ = 0.17, F(1,42) = 10.20, *p* < 0.01; β = -17.22).

58% of OA experienced vection during the RDT compared to 87% in the YA group, with YA showing higher average vection scores (OA: 0.9 ± 1.6 vs. YA: 1.6 ± 1.1; U = 272.5; *p* = 0.03; Fig. 3F). Nausea induced by the RDT was experienced by 16% of OA and 15% of YA, with no difference in mean scores between the two groups (OA: 0.1 ± 0.2; YA: 0.2 ± 0.5; U = 193; *p* = 0.98).

There were no differences in motion sickness susceptibility (OA: 14.5 ± 11.3 vs. YA: 11.7 ± 10.2; U = 162.5; *p* = 0.44), and situational vertigo scores (OA: 0.34 ± 0.43 vs. YA: 0.23 ± 0.27; U = 166.5; *p* = 0.55). However, there was a significant association between motion sickness susceptibility and LPV (cm; R^2^ = 0.15, F(1,42) = 8.57, *p* < 0.01; β = -0.18).

### Relationship with SVV tilt error

**Fig. 4:**
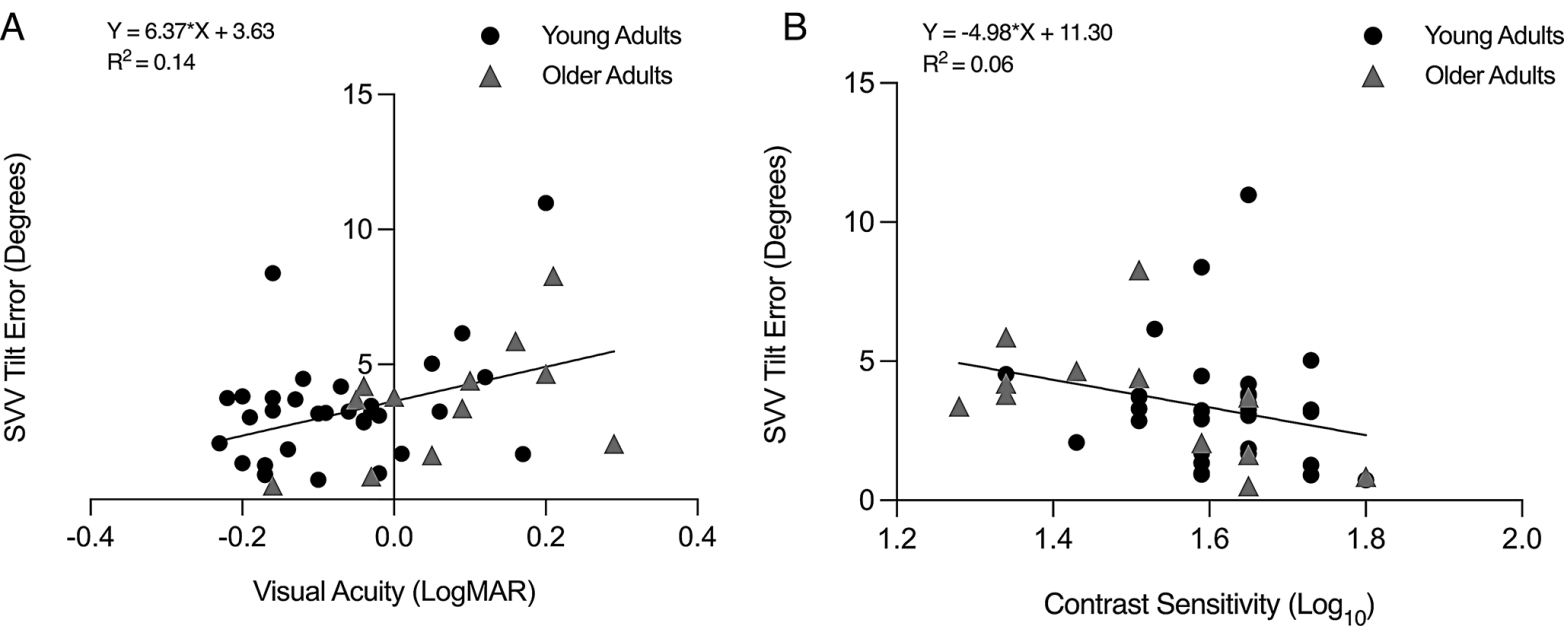
Multiple linear regression analysis between visual function and visual dependence (SVV tilt error in degrees). **A**) Visual Acuity (LogMAR) vs. SVV tilt errors, and **B**) Contrast Sensitivity (Log_10_) vs. SVV tilt error

A linear regression model found that age was not a predictor of variability in SVV (β = 0.27, *p* = 0.71). However, there were significant associations between SVV tilt error and visual acuity (R^2^ = 0.14, F(1,42) = 8.00, *p* < 0.01; β = 6.37; Fig. 4A), contrast sensitivity (CS; R^2^ = 0.07, F(1,42) = 3.93, *p* = 0.03; β = −4.97; Fig. 4B), and vection (R^2^ = 0.19, F(1,42) = 10.91, *p* < 0.01; β = 0.75). Multiple linear regression analysis revealed that visual acuity (VA) (β = 9.63, *p* < 0.01) significantly predicted SVV tilt error, whilst contrast sensitivity (CS) (β = -3.04, *p* = 0.25), lower visual field (β = 0.006, *p* = 0.59), and age (β = -1.31, *p* = 0.09) were not significant predictors. Depth perception tended to have a small but significant negative relationship with visual dependence (β = -2.95, *p* = 0.04). Overall, the model explained 23% of the variance in SVV tilt error (R² = 0.23, F(5,38) = 3.51, *p* = 0.01).

### Mediation analysis

**Fig. 5:**
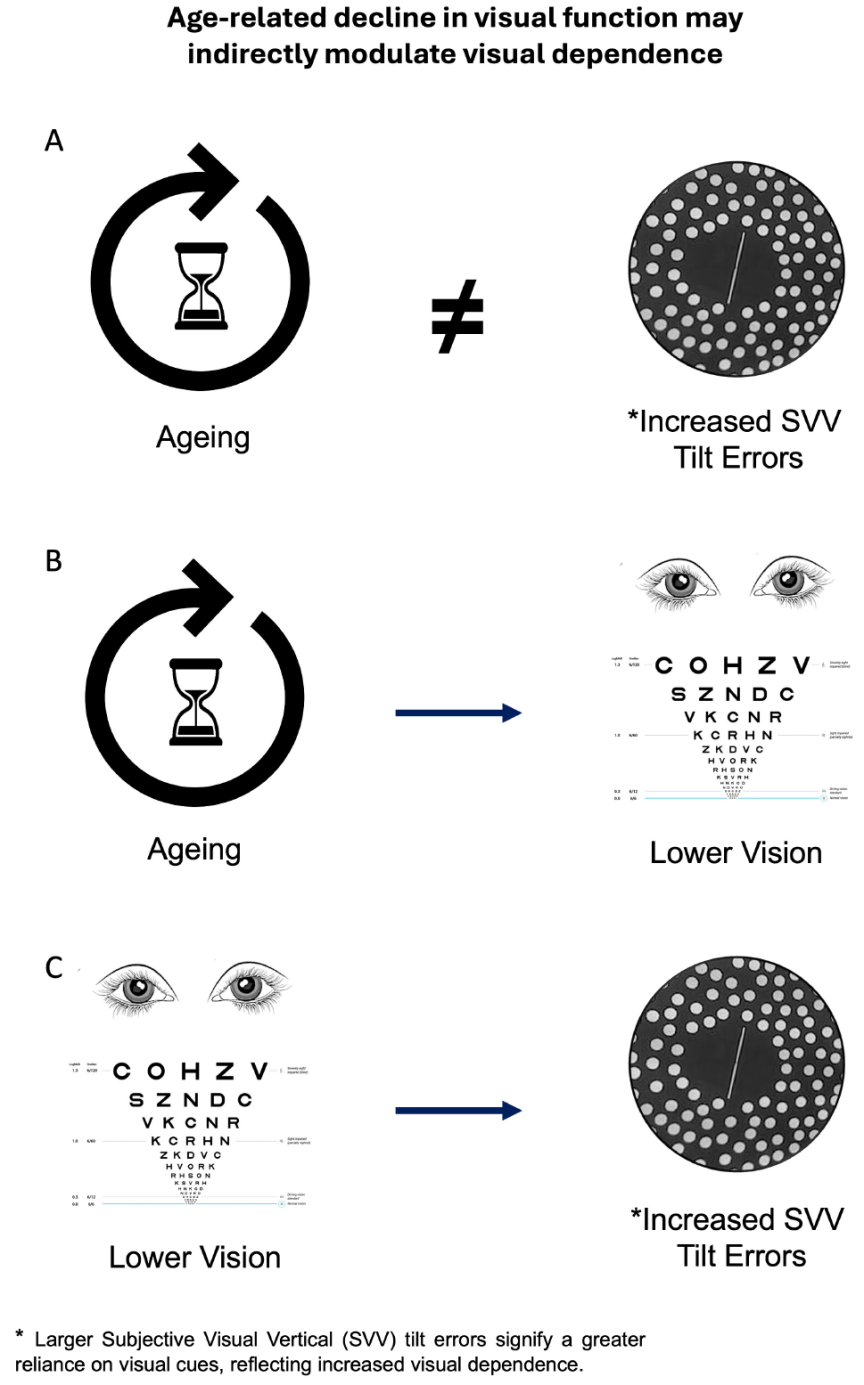
Visual abstract of key findings. **A**) SVV tilt errors were not associated with ageing, suggesting that age alone is not a predictor of visual dependence in this cohort. **B**) Visual function, specifically Visual Acuity (VA) and Contrast Sensitivity (CS), decline with age. **C**) Lower VA and CS are associated with increased SVV tilt errors; this suggests that variations in visual function, rather than age, may modulate visual dependence in this cohort

Visual acuity (VA; LogMAR) and contrast sensitivity (CS; log CS) that independently accounted for small but significant visual dependence variability were included in ACME analysis. There was no Average Direct Effect (ADE) of age on SVV (*p* = 0.37), however ACME, representing the indirect effect of age on SVV, was significant for both VA (*b* = 1.08, 95% CI [0.25, 2.09], *p* = 0.03) and CS (*b* = 0.55, 95% CI [0.01, 1.43], *p* = 0.04). This suggests that the effect of age on SVV is primarily mediated through its impact on VA and, to a lesser extent, CS Fig. 5.

## Discussion

Our main findings were that healthy young and older adults had similar SVV tilt error, and thus visual dependence. However, visual acuity and contrast sensitivity, which were observed to be lower in the older adults, were associated with greater SVV tilt error. ACME analysis identified significant modulation of SVV tilt error via age-related declines in both visual acuity and contrast sensitivity. In contrast, depth perception and lower peripheral vision did not differ between the groups. Depth perception, however, had a small but significant negative relationship with SVV. Vision-related health and quality of life (VFQ-25) were similar in each group, although higher VFQ-25 scores were associated with better visual acuity. Young adults reported higher RDT-induced vection, however, there were no differences in nausea, VMSQ or MSSQ scores between the groups.

### Decline in visual function with age

The observation of lower visual acuity and contrast sensitivity in older adults is consistent with previous research (Saftari and Kwon 2018b, a). Notably, our cohort possessed clinically good (corrected or habitual) visual acuity (LogMAR < 0.3). The finding that depth perception did not decline with age is consistent with prior research (Norman et al. 2008). However, whilst we employed the classical Howard-Dolman apparatus method, there are other more modern techniques such as the Randot test (Wang et al. 2010).

In contrast to previous research, we did not observe an age-related decline in lower peripheral vision (Ramrattan et al. 2001). This failure may relate to the fact that our participants, regardless of age, were free from ophthalmological conditions such as glaucoma and macular degeneration, which are associated with peripheral vision loss (Ramrattan et al. 2001). *The LPV test in this study*,* whilst inexpensive and practical*,* lacked the precision and granularity of automated visual field analysers such as the Goldmann perimetry apparatus* (Broadway 2012). *As a result*,* it is possible that subtle declines in the lower visual fields were not detected. Future studies*,* particularly in healthy adults could benefit from incorporating more sensitive automated perimetry techniques to detect sub-clinical changes in peripheral vision.*

### Subjective assessment of visual function is associated with ageing

Older adults demonstrated marginally (sub-clinical) lower visual acuity (VA) and contrast sensitivity (CS); however, their self-perceived vision-related health and quality of life were comparable to younger adults. Older adults in our study reported higher VFQ-25 scores (91.6 ± 6.3) compared to those (54.4 ± 21.5) reported by Inoue and colleagues, although their participants (69.6 ± 14.5 yrs) were older than those in our study (62.4 ± 6.7 yrs) (Inoue et al. 2018).

Additionally, our participants were healthy, physically (albeit not formally assessed) and socio-economically active, and free from major co-morbidities and ophthalmological pathology. Control of these factors may explain the failure to observe differences in VFQ-25 in our cohort. However, the VFQ-25 may be a useful proxy instrument in those with low level, but functionally significant visual pathology – although an issue may be that older adults may recalibrate expectation and satisfaction (Cho and Cheon 2023). In future, concurrent assessment of other perceptual domains associated with vestibular and/or proprioceptive pathology may be warranted.

### No association between visual dependence and ageing

In our study, age was not an independent predictor of SVV tilt error. This finding contrasts with previous research, which attribute increased visual dependence to age-related declines in non-visual sensory and sensorimotor function (Osoba et al. 2019b). In fact, SVV tilt error was comparable between our older and younger adults. This is consistent with that reported by Lee (Lee 2017), who (albeit in a small sample) also observed significant inter-observer variability in SVV tilt error among older adults.

In contrast, Agathos and colleagues (Agathos et al. 2015a), reported an age-related increase in SVV tilt error (+ 4.9 ± 2.0 degrees) in a cohort of twenty older adults. This cohort was larger than our older adult group (*n* = 12) but also critically it was significantly older (74.1 ± 3.7 vs. 62.4 ± 6.7 years). Thus, our group is likely to present less significant multi-systems degradation.

Furthermore, we recruited healthy individuals with active lifestyles, as older adults who remain physically active exhibit superior proprioceptive (Ribeiro and Oliveira 2007), vestibular (Wiszomirska et al. 2015), and cognitive function (Xu et al. 2023) with age. Given higher levels of visual dependence in older adults is proposed to result from impaired sensory and/or sensorimotor function, it is plausible that in addition to accounting for differences between cohorts, physical activity levels in older adults may relate to intra-individual variation in visual dependence in older adults – both reflecting and driving MSI.

Unfortunately, we were unable to quantify habitual physical activity or relevant cognitive function (Green et al. 2010). However, older active ex-tennis professionals have previously demonstrated lower visual dependence than sedentary controls (Rotella and Bunker 1978). Thus, evaluation of activity as a co-factor with sensory function and their association with visual dependence in older adults warrants further study.

### Increased visual dependence is associated with clinically insignificant visual function deficits

Multiple regression modelling identified sub-clinical variability in visual acuity as the primary predictor of SVV tilt error variability, whilst depth perception demonstrated a small but significant (negative) relationship with SVV. Contrast sensitivity was also associated with increased SVV tilt error, although only at the univariate level. Agathos and colleagues (Agathos and Shanidze 2024) reported correlations between visual acuity, contrast sensitivity and SVV tilt error in individuals with central field loss - noting that contrast sensitivity had a stronger association.

One potential explanation for the fact that in our study visual acuity was the stronger predictor is that whilst contrast sensitivity levels were similar between the two studies (1.43 ± 0.26 vs. 1.51 ± 0.16; 94.7% agreement; Cohen’s d = 0.81), visual acuity (LogMAR) was significantly reduced in Agathos’s study (0.80 ± 0.60 vs. 0.07 ± 0.13; 8.7% agreement; Cohen’s d = -1.90) – reflective of their CFL.

Whilst there was no direct effect of ageing on visual dependence, ACME analysis indicates that the association between visual dependence, and both lower visual acuity and contrast sensitivity represents an indirect corollary of ageing. The paradox of why some older adults rely on visual information, despite declining visual function is unresolved. However, significant SVV tilt error inter-individual variability suggests that whilst visual signal processing is important – there are likely to be a range of factors (Osoba et al. 2019a) that contribute to the tendency to prioritise visual signals. Thus, subsequent studies should seek to quantify multi-sensory function (including visual, vestibular and proprioceptive), cognitive function, perception of function and falls risk along with physical activity in healthy and unisensory pathological groups to ascertain inter-relationships via ACME analysis.

On potential issue with the use of the RDT to sensitively assess visual dependence is its dependence upon having sufficient visual fidelity to not differently affect the assessment of the fluorescent rod position, and thus tilt. Despite this issue the test has been used even with visual pathology (Agathos and Shanidze 2024). In our study, since most participants demonstrated normal visual acuity (The Royal College of Ophthalmologists 2021), it is unlikely that sub-clinically ‘lower’ vision limited their performance. However, subsequent studies evaluating a greater range of visual fidelity, to confirm that this was not a limiting factor, should be performed by calculating the angular separation of adjacent dots within the RDT interface. Such data may even warrant being a feature of RDT testing. Additionally, comparisons with other methods of visual dependence testing (e.g., VIRVEST (Totilienė et al. 2021) warrant consideration.

### Vision, visual dependence, and the law of inverse effectiveness

The observation that lower sub-clinical visual function (visual acuity and contrast sensitivity) was associated with increased SVV tilt error and hence visual dependence, challenges the sensory reweighting theory (Peterka 2002), which describes the downregulation of lower fidelity sensory signals, and upweighting of higher fidelity signals. The design of the RDT test attempted to minimize vestibular and proprioceptive feedback, however, they may have still played a role – particularly as our participants were recruited with no evident sensory dysfunction. Whether the prioritisation of ‘poorer’ visual afferents is concurrent with the relative degradation of vestibular and proprioceptive signals, warrants further study.

Alternatively, the ‘law of inverse effectiveness’ (Stevenson et al. 2012), posits that multisensory integration is more effective when individual sensory inputs are weak or ambiguous. Thus, by minimising vestibular and proprioceptive feedback we may exaggerate visual dependence. Whilst this is a trade-off, this approach was more ecological than Romberg testing with eyes closed (Black et al. 1982).

### Increased vection is associated greater visual dependence

Higher SVV tilt error was associated with higher vection rating consistent with previous studies (Pavlou et al. 2011). However, were no differences in RDT-induced nausea, VMSQ scores, or susceptibility to motion sickness between younger and older adults. Vection did not relate to any visual function parameters, but further exploration is warranted with groups with higher motion sickness susceptibility – which was low in our groups. This low susceptibility also accounts for the absence, or only low levels nausea in response to the RFT.

Interestingly, susceptibility to motion sickness was associated with better lower peripheral vision – which itself was similar in each group. Peripheral vision is crucial for detecting motion and maintaining spatial orientation. The independent findings of higher vection scores in visually dependent individuals, and increased susceptibility to motion sickness in individuals with better lower peripheral vision, may indicate a greater reliance on peripheral visual cues for orientation. Despite there being no interaction between visual acuity, contrast sensitivity and lower peripheral vision in this study, it is plausible, as suggested by Agathos and colleagues (Agathos and Shanidze 2024), that in individuals with poorer central field vision, peripheral visual cues become more pertinent for spatial orientation. Whether these findings persist in larger cohorts require studies with differential visual fidelities within the clinical, and as in this study the sub-clinical range, along with physical activity levels, given that visual impairment is associated with limitation of physical activity and fear of falling(Nguyen et al. 2015).

## Conclusion

Subclinical differences in visual acuity and contrast sensitivity contributed to SVV tilt error variability, among both healthy young and older adults. Thus, whilst ageing was not independently associated with increased SVV tilt error – and thus visual dependence – as older adults exhibited relatively poorer visual acuity and contrast sensitivity, this accounted for modest modulation of SVV tilt error. This was despite subjective vision-related quality of life being similar between young and older adults. Young adults reported higher RDT-induced vection, however, there were no differences in nausea, in line with generalised susceptibility to motion sickness between groups. Therefore, aspects of visual function fidelity may play a key role in visual dependence, rather than age per se. Further studies are needed to define the inter-relationships between age-related visual function, non-visual factors (including vestibular and proprioceptive fidelity, activity levels, fear of falling and cognitive function) and visual dependence.

## Data Availability

The data that support the findings of this study are not openly available due to reasons of sensitivity and are available from the corresponding author upon reasonable request.
